# Supraoptimal Brassinosteroid Levels Inhibit Root Growth by Reducing Root Meristem and Cell Elongation in Rice

**DOI:** 10.3390/plants10091962

**Published:** 2021-09-20

**Authors:** Kewalee Jantapo, Watcharapong Wimonchaijit, Wenfei Wang, Juthamas Chaiwanon

**Affiliations:** 1 Center of Excellence in Environment and Plant Physiology, Department of Botany, Faculty of Science, Chulalongkorn University, Bangkok 10330, Thailand; k.jantapo@gmail.com (K.J.); watcharapong.wmcj@gmail.com (W.W.); 2 Program in Biotechnology, Faculty of Science, Chulalongkorn University, Bangkok 10330, Thailand; 3 College of Life Sciences, Fujian Agriculture and Forestry University, Fuzhou 350002, China; wenfeiwang@fafu.edu.cn

**Keywords:** brassinosteroid, propiconazole, nitrogen deficiency, root meristem, rice

## Abstract

Root growth depends on cell proliferation and cell elongation at the root meristem, which are controlled by plant hormones and nutrient availability. As a foraging strategy, rice (*Oryza sativa* L.) grows longer roots when nitrogen (N) is scarce. However, how the plant steroid hormone brassinosteroid (BR) regulates rice root meristem development and responses to N deficiency remains unclear. Here, we show that BR has a negative effect on meristem size and a dose-dependent effect on cell elongation in roots of rice seedlings treated with exogenous BR (24-epicastasterone, ECS) and the BR biosynthesis inhibitor propiconazole (PPZ). A genome-wide transcriptome analysis identified 4110 and 3076 differentially expressed genes in response to ECS and PPZ treatments, respectively. The gene ontology (GO) analysis shows that terms related to cell proliferation and cell elongation were enriched among the ECS-repressed genes. Furthermore, microscopic analysis of ECS- and PPZ-treated roots grown under N-sufficient and N-deficient conditions demonstrates that exogenous BR or PPZ application could not enhance N deficiency-mediated root elongation promotion as the treatments could not promote root meristem size and cell elongation simultaneously. Our study demonstrates that optimal levels of BR in the rice root meristem are crucial for optimal root growth and the foraging response to N deficiency.

## 1. Introduction

Root systems play important roles in water and nutrient acquisition. The developmental plasticity of root system architecture is crucial for crop adaptation to unfavorable environments, such as drought stress and nutrient deficiency stress. For example, rice varieties with larger root biomass, a more extensive root distribution, and a longer root length were found to use nitrogen more efficiently [1]. Understanding the mechanisms that control root growth is important for crop genetic improvement for sustainable agriculture, with the goal of reducing fertilizer application while maintaining crop productivity.

Root growth is determined by cell division and elongation at the root tip, where cells are organized along the longitudinal axis in distinct developmental zones. At the apical side of the root tip, cells are actively dividing in the meristem zone. As the cells leave the meristem zone, they enter the elongation zone, where they rapidly elongate and reach their mature size before entering the maturation zone to undergo differentiation [2]. Optimal root growth thus depends on the root meristem size and the number of dividing meristematic cells [3]. Several studies have demonstrated that root growth and meristem size are regulated by several internal and external factors, including plant hormones and nutrient availability in the soil [4].

Brassinosteroid (BR) is a class of steroid hormones that regulates various physiological processes such as hypocotyl cell elongation, photomorphogenesis, and stomatal development [5]. BR regulates rice and Arabidopsis root growth in a dose-dependent manner with low BR concentrations marginally promoting root growth and high BR concentrations dramatically inhibiting root growth [6,7]. Several BR and their roles in root meristem development have been studied using Arabidopsis roots as a model. Enhanced BR signaling results in premature cell cycle exit, and inhibits root meristem size in Arabidopsis [8]. In addition, previous transcriptomic analysis showed that BR promoted expression of cell elongation-related genes expressed in the root transition-elongation zone, but repressed several genes specifically expressed in the meristem zone [6].

Nitrogen (N) is one of the most important macronutrients for plant growth and development, and it is frequently a key limiting factor in most agricultural systems. When grown under N deficiency, plants exhibit root foraging responses with increased root length, which allows them to explore more soil volumes to improve N uptake ability [9,10]. BR has been shown to regulate root adaptation responses to various nutrient deficiency including N, phosphorus, iron and boron [11]. Works in Arabidopsis have demonstrated that a natural allelic variation in a BR signaling component, BSK3, which leads to enhanced sensitivity of the BR signaling pathway, as well as upregulation of BR biosynthesis in roots, could promote primary root elongation under mild N deficiency [12,13]. However, it remains unclear how BR regulates rice root meristem development and its responses to N deficiency.

In this study, we examined the effect of 24-epicastasterone (ECS) and propiconazole (PPZ), a BR biosynthesis inhibitor [14], on root meristem size and cell elongation in rice seedlings grown under N-sufficient and N-deficient conditions. Furthermore, we performed an RNA-sequencing analysis to identify ECS- and PPZ-responsive genes in the root under N-sufficient condition. Our results show that supraoptimal BR levels inhibited root cell proliferation and elongation, as well as expression of genes involved in cell proliferation and cell elongation and that optimal BR levels were crucial for N deficiency-induced root growth promotion.

## 2. Results

### 2.1. Effect of BR on Root Elongation

**Figure 1:**
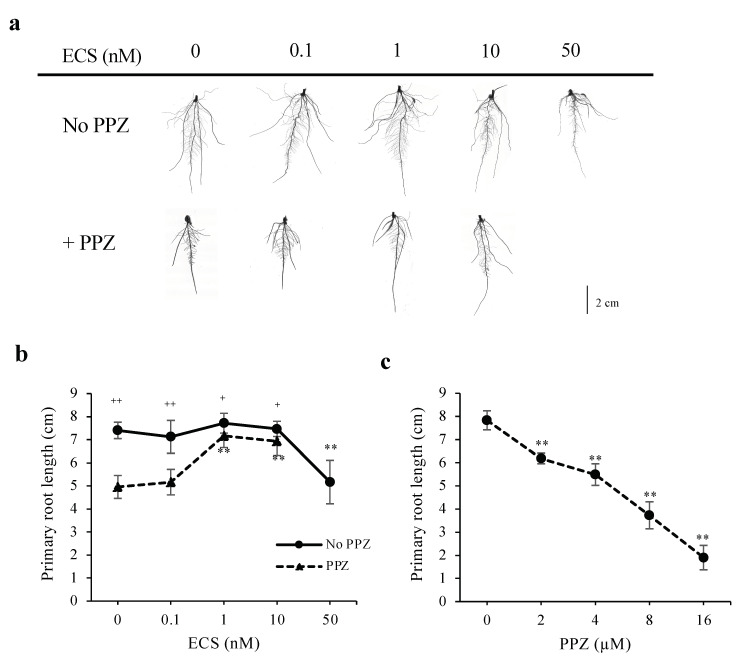
Effect of ECS and PPZ treatments on rice root growth. Root phenotypes of rice seedlings grown for 5 d under different concentrations of ECS or PPZ or combination of ECS and PPZ (4 μM). (**a**) Representative images of roots grown under different treatments. Scale bar = 2 cm. (**b**,**c**) Quantification of primary root length. Data are means ± SD (*n* = 10 biological replicates). Significant differences between the treatment and the mock control are indicated by ** for *p* < 0.001. Significant differences between PPZ and no PPZ (with the same ECS concentration) are indicated by ^+^ and ^++^ for *p* < 0.05 and 0.001, respectively.

To investigate how BR modulates root growth, germinated rice seeds were grown for 5 days (d) in media supplemented with various concentrations of a biologically active BR, 24-epicastasterone (ECS), and/or a BR biosynthesis inhibitor, propiconazole (PPZ). Treatments of ECS at concentrations up to 10 nM did not change primary root length, whereas 50 nM ECS inhibited root length significantly (Figure 1a,b). Increased PPZ concentrations resulted in more reduction in primary root length (Figure 1c). The inhibitory effect of 4 μM PPZ on primary root length could be rescued by 1 nM and 10 nM ECS (Figure 1a,b). These results suggest that the effect of BR on root elongation is dose-dependent.

### 2.2. Effect of BR on Root Cell Proliferation and Elongation

**Figure 2:**
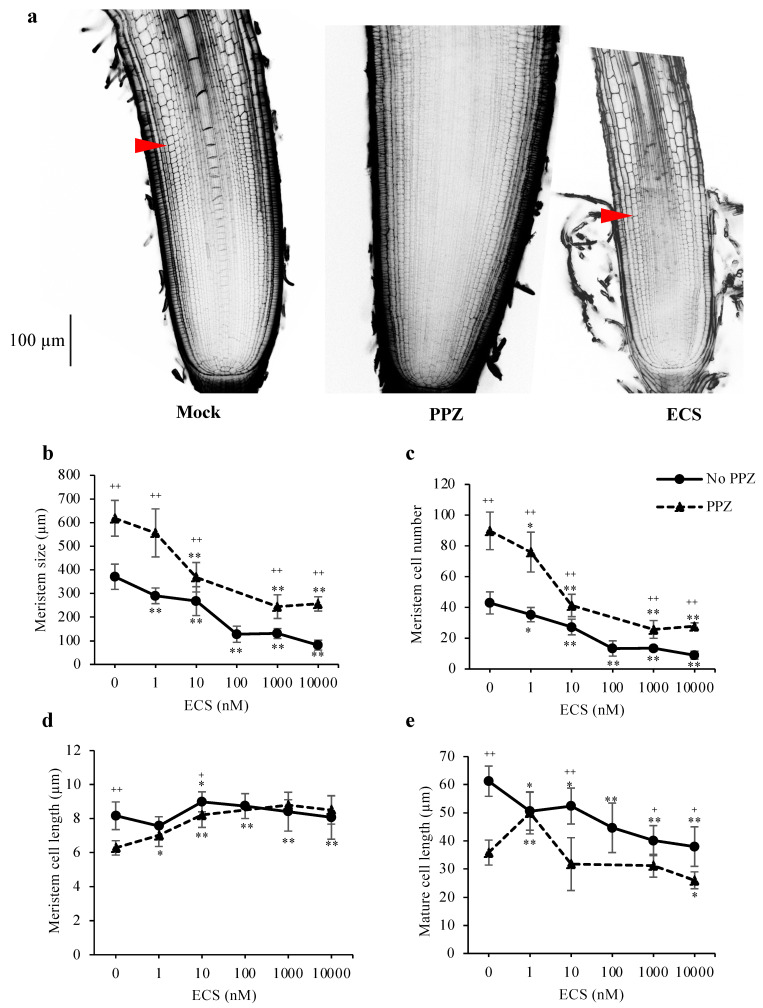
Effect of ECS and PPZ treatments on rice root meristem and cell elongation in primary root tips. Seedlings were grown in the absence and presence of PPZ for 5 d, and then treated with ECS for 24 h. (**a**) Confocal microscopy images of rice root meristems treated with mock, PPZ (4 μM) or ECS (10 nM). Scale bar = 100 μm. Arrowheads mark the end of the meristem zone; the PPZ-treated root had large meristem that the end of the meristem zone was not present in the image. (**b**–**d**) Quantifications of root meristem size (**b**), meristem cell number (**c**) and average meristem cell length (**d**) were determined from cortical cells in the 4th cortical layer by measuring from the QC to the first elongated cell. Mature cell length (**e**) was determined from the average length of five adjacent mature cortical cells. Data are means ± SD (*n* ≥ 6 biological replicates). Significant differences between the treatment and the mock control are indicated by * and ** for *p* < 0.05 and 0.001, respectively. Significant differences between PPZ and no PPZ (with the same ECS concentration) are indicated by ^+^ and ^++^ for *p* < 0.05 and 0.001, respectively.

To understand how BR regulates cell proliferation and elongation in the root apices, primary root tips of seedlings grown in the presence or absence of PPZ were treated with ECS for 24 h and observed under microscope. Quantification of cell number and cell length in the 4th cortical layer of the root meristem showed that PPZ treatment increased root meristem size and meristem cell number, but reduced cell length (Figure 2a–d). ECS treatments reduced meristem size and meristem cell number in a dose-dependent manner under both PPZ and no PPZ conditions. Treatment of 10 nM ECS for 24 h could restore meristem size, meristem cell number and cell length of PPZ-treated roots to those of the untreated control (Figure 2b–d). Higher concentrations of ECS strongly reduced meristem size and meristem cell number but did not further increase meristem cell length (Figure 2b–d). This result suggests that BR had a negative effect on root meristem size and meristem cell number and a positive effect on meristem cell elongation.

In addition to meristem cell proliferation, cell elongation in the elongation zone, which determines mature cell length, also contributes to root elongation rate. ECS treatment significantly reduced mature cell length. PPZ treatment also reduced mature cell length to 59% of the untreated control, which could be partially rescued by 1 nM ECS (Figure 2e). However, higher concentrations of ECS could not promote cell elongation inhibited by PPZ (Figure 2e). The results show that BR had a dose-dependent effect on root cell elongation, with low concentrations promoting cell elongation and high concentrations inhibiting it.

### 2.3. Transcriptome Profiling of ECS- and PPZ-Treated Rice Roots

**Figure 3:**
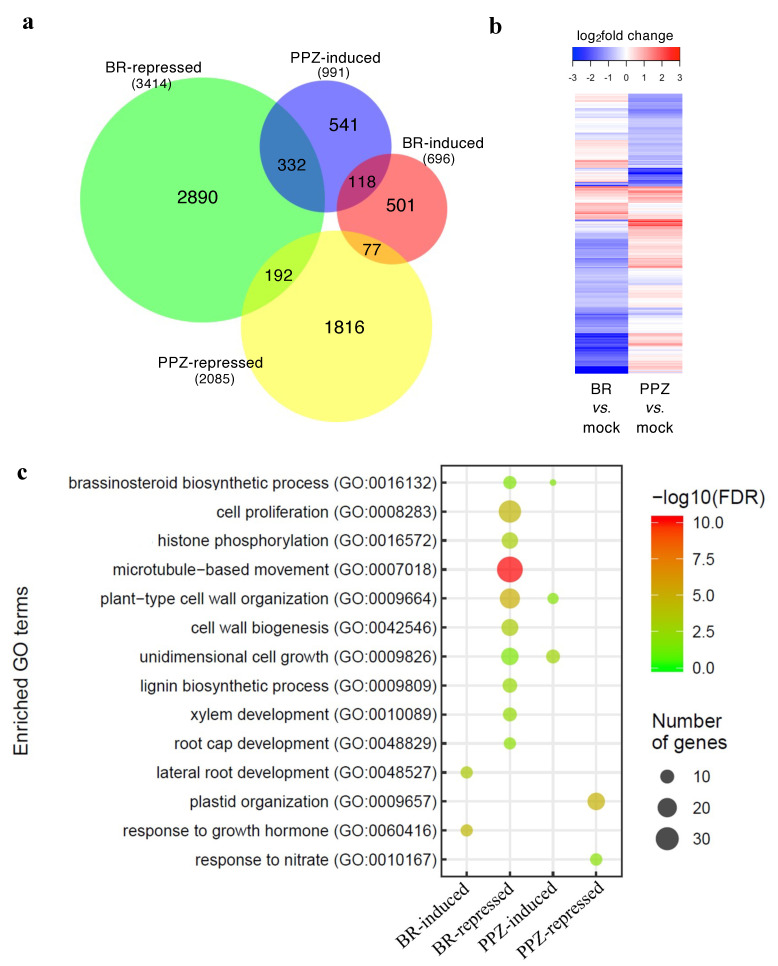
Transcriptomic analysis of differentially expressed genes in ECS- and PPZ-treated rice roots. (**a**) Venn diagram showing the overlap between the lists of significant ECS-induced, ECS-repressed, PPZ-induced and PPZ-repressed genes (|fold change| > 1.5; adjusted *p*-value < 0.05). The numbers of DEGs are shown in parentheses. (**b**) Hierarchically clustered heatmap displaying the log_2_FC values of all significant genes in the ECS vs. mock or PPZ vs. mock comparisons. (**c**) GO biological process term enrichment analysis of the ECS and PPZ DEG lists.

To understand how high and low (physiological) concentrations of BR regulate root elongation at transcriptional levels, we performed transcriptomic analysis of roots treated with a high concentration of ECS for 24 h (+ECS), or grown in media supplemented with PPZ (+PPZ) or without PPZ (mock). Expression profiles of ECS- and PPZ-treated samples were compared with the mock control (+ECS vs. mock and +PPZ vs. mock) to identify ECS- and PPZ-responsive genes, respectively. Genes that were significantly differentially expressed by more than 1.5 folds (|log_2_fold change| > 0.58 and adjusted *p*-value < 0.05) were included in the differentially expressed gene (DEG) list for further analysis. ECS treatment induced 696 genes and repressed 3414 genes, whereas PPZ treatment induced 991 genes and repressed 2085 genes (Figure 3a, Table S1). Venn diagram and heatmap clustering of DEGs show that about one-third of the PPZ-induced genes were also repressed by ECS, while there was little overlap between PPZ-repressed genes and ECS-induced or ECS-repressed genes.

Gene ontology (GO) enrichment analysis identified significantly enriched GO terms (biological process) among the ECS-repressed DEGs related to cell proliferation and cell elongation including ‘cell proliferation’, ‘histone phosphorylation’, ‘microtubule-based movement’, ‘plant-type cell wall organization’, ‘cell wall biogenesis’ and ‘unidimensional cell growth’ (Figure 3c). The terms ‘lignin biosynthetic process’, ‘xylem development’ and ‘root cap development’ were also enriched among the ECS-repressed DEGs, whereas the terms ‘lateral root development’ and ‘response to growth hormone’ were enriched among the ECS-induced DEGs. The terms ‘plastid organization’ and ‘response to nitrate’ were enriched among the PPZ-repressed DEGs.

**Figure 4:**
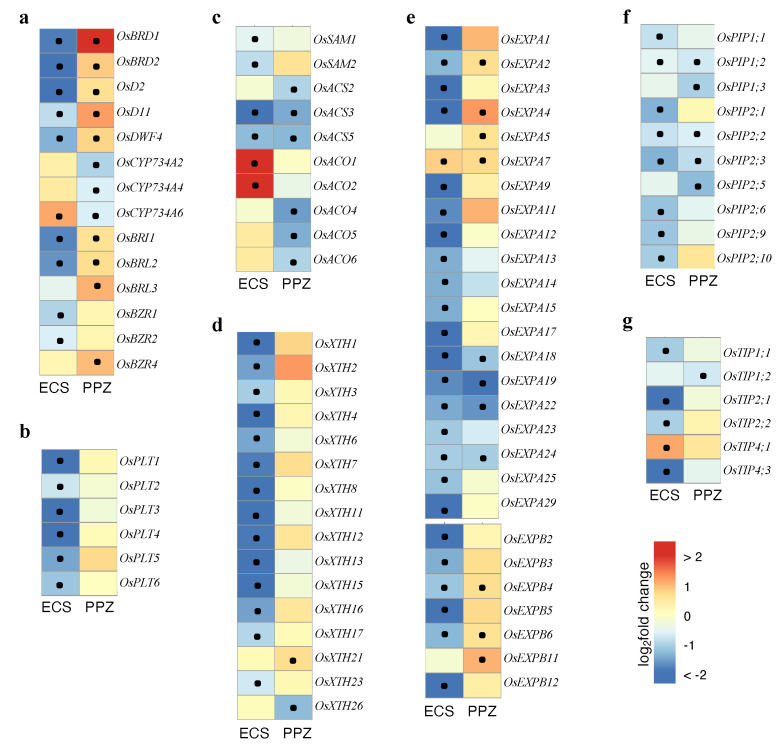
Expression of genes involved in BR biosynthesis and signaling, ethylene biosynthesis, cell proliferation and cell elongation. Heatmaps represent log2FC values of genes in the ECS vs. mock or PPZ vs. mock comparisons. Black dots indicate statistical significance of differential expression (adjusted *p*-value < 0.05). (**a**) BR biosynthetic and signaling genes, (**b**) *OsPLT* genes, (**c**) ethylene biosynthesis genes, (**d**,**e**) cell wall loosening and remodeling genes *OsXTHs* (**d**) and *OsEXPs* (**e**), (**f**,**g**) aquaporin genes *OsPIPs* (**f**) and *OsTIPs* (**g**). Only genes that showed statistical significance in at least one of the ECS or PPZ comparisons were included in this figure.

BR biosynthetic genes (*OsBRD1, OsBRD2, OsD2, OsD11* and *OsDWF4*) were repressed by ECS and induced by PPZ, whereas BR catabolic genes (*OsCYP73A2, OsCYP73A4* and *OsCYP73A6*) were induced by ECS and repressed by PPZ (Figure 4a). In addition, ECS repressed and PPZ induced expression of BR receptor genes (*OsBRI1, OsBRL2* and *OsBRL3*) and BZR family transcription factors (*OsBZR1, OsBZR2* and *OsBZR4*), with the exception of *OsBZR4*, which was induced by ECS (Figure 4a). The expression of these BR biosynthetic, catabolic and signaling genes, which showed negative feedback regulation by the BR signaling pathway [15], corroborated that BR signaling was activated in the ECS-treated roots and inhibited in the PPZ-treated roots.

The PLETHORA (PLT) family transcription factors are known to be master regulators of root meristem size [16]. Among 10 *OsPLT* genes identified in rice, *OsPLT1-6*, which are expressed in rice roots [17], were all repressed by ECS (Figure 4b). This result suggests that ECS inhibited root meristem size partly by repressing *OsPLT* expression. ECS also repressed several microtubule-related genes, which are involved in cell division [18]. These included 9 out of 12 tubulin genes, 27 out of 52 kinesin genes and 5 out of 11 microtubule-associated protein 65 (MAP65) genes identified in rice (Table S2).

Ethylene is another plant hormone that has been known to inhibit root elongation [19]. Ethylene biosynthesis is catalyzed by the enzymes S-adenosylmethionine (SAM) synthase, 1-aminocyclopropane-1-carboxylate (ACC) synthase (ACS), and ACC oxidase (ACO) [20]. We found that *OsACO1* and *OsACO2* were among the four strongest ECS-induced genes (log_2_fold change = 2.60 and 2.23, respectively; Table S1), while *OsACO4*, *OsACO5* and *OsACO6* were significantly repressed by PPZ (Figure 3c), suggesting that ECS treatments may potentially increase ethylene contents by upregulating *OsACO* expression. However, genes encoding *OsSAMS1/2* and *OsACS2/3/5* were repressed by ECS and/or PPZ.

Expansins (EXP) and xyloglucan endotransglucosylase/hydrolase (XTH) play important roles in cell wall loosening and remodeling, thus mediating root cell elongation. We found that ECS treatment repressed 18 *OsEXPA*s, 6 *OsEXPBs* and 14 *OsXTHs*. PPZ treatment repressed 4 *OsEXPA*s and 1 *OsXTH*, but induced 3 *OsEXPAs,* 3 *OsEXPBs* and 1 *OsXTH* (Figure 4d,e). In addition, ECS and PPZ repressed expression of aquaporins, including plasma membrane intrinsic proteins (PIPs) and tonoplast intrinsic proteins (TIPs), which are important regulators of osmotic water transport, cell turgor pressure and cell elongation [21]. ECS significantly repressed eight *OsPIPs* and four *OsTIPs*, while PPZ repressed five *OsPIPs* and one *OsTIP.* Only *OsTIP4;1* was significantly induced by ECS (Figure 4f,g). These results suggest that a high concentration of BR inhibited root cell elongation by downregulating expression of most cell wall-loosening and remodeling enzymes and aquaporins. However, limited cell elongation caused by PPZ treatment is most likely due to reduced expression of aquaporins and certain expansins. The negative effect of high concentrations of ECS on cell proliferation and cell elongation-related genes suggests that optimal BR level in the root is critical for optimal root elongation.

### 2.4. Effect of BR on N Deficiency-Induced Root Elongation

**Figure 5:**
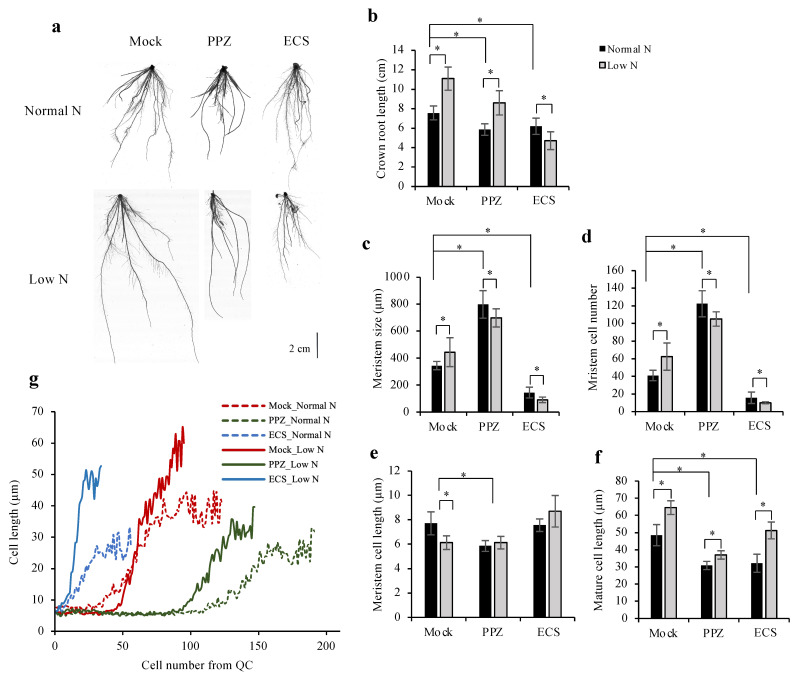
Effect of ECS and PPZ treatments on root growth responses to N deficiency. Germinated seeds were grown in normal N for 5 d and then transferred to either normal N or low N conditions containing 10 nM ECS or 4 μM PPZ or mock for 7 d. (**a**) Representative images of roots grown under different treatments. Scale bar = 2 cm. (**b**) Crown root length was calculated from the average of the three longest crown roots. Data are means ± SD (*n* = 10 biological replicates). (**c**–**g**) Quantifications of root meristem size (**c**), meristem cell number (**d**) and average meristem cell length (**e**) in the crown roots were determined from cortical cells in the 4th cortical layer by measuring from the QC to the first elongated cell. Mature cell length (**f**) was determined from the average length of five adjacent mature cortical cells. (**g**) Average cortical cell length along the longitudinal root axis from the QC illustrated the number of cells in the meristem, the onset of rapid cell elongation and the effect of low N on promoting cell elongation under mock and ECS treatments but not PPZ treatment. Data are means ± SD (*n* ≥ 6 biological replicates). Significant differences are indicated by * for *p* < 0.05.

To study how increased or decreased BR levels affect low N-mediated root elongation promotion, germinated seeds were grown in N-sufficient conditions (normal N) for 5 d and then transferred to either normal N or low N conditions, which were supplemented with 10 nM ECS or 4 μM PPZ or mock. After 7 d of treatment, crown roots were used to measure growth and root meristems because the primary root of monocots dies as the plants age and had stopped growing in our experiment. Under low-N conditions, mock- and PPZ-treated roots had longer crown roots than normal N-treated roots, while ECS-treated roots had shorter roots (Figure 5a,b). Low N increased root meristem size, meristem cell number and mature cell length while decreasing meristem cell length (Figure 5c-f). PPZ-treated roots had a larger meristem size than mock-treated roots, and low N did not increase it any further (Figure 5c). PPZ treatment, on the other hand, reduced the promoting effect of low N on mature cell length (Figure 5f), suggesting that endogenous BR is involved in root cell elongation in response to N deficiency.

Interestingly, ECS-treated roots were more strongly inhibited under low N compared to normal N conditions. In ECS-treated roots, low N treatment reduced meristem size and meristem cell number but allowed promotion of mature cell length (Figure 5c,d,f). Measurement of cell length in the 4th cortical layer along the longitudinal root axis demonstrates the point of transition into the elongation zone, where cells rapidly increase their length. Figure 5g shows that ECS treatment caused premature cell cycle exit and that low N + ECS treatment enhanced cell elongation of the elongating cells, consistent with the further reduction in root meristem size (Figure 5c,d) and root length (Figure 5b).

## 3. Discussion

Root length is a critical factor for crop productivity, as deeper roots can potentially increase soil exploration for water and nutrient uptake [22]. Optimal root growth depends on the size of root meristem, which is controlled by the balance between cell proliferation and cell elongation along the root developmental zones [23]. Here, we demonstrated that BR has a negative effect on meristem size and a dose-dependent effect on cell elongation in rice roots. Transcriptome analysis showed consistently that a high concentration of BR downregulated cell proliferation- and cell elongation-related genes. We then demonstrated that exogenous BR or PPZ application could not enhance root elongation promotion by N deficiency as the treatments could not simultaneously promote root meristem size and mature cell length.

### 3.1. Effect of BR and PPZ on Rice Root Meristem Cell Proliferation and Cell Elongation

BR has been shown to negatively regulate Arabidopsis root meristem size by promoting cell elongation and accelerating cell cycle exit [8]. As shown here, PPZ-treated roots exhibited reduced cell elongation and a large meristem size, likely due to a delay in cell cycle exit. Our results reveal that low concentrations of ECS could promote root cell elongation of the PPZ-treated meristematic and mature cells (Figure 2d,e), consistent with the well-known function of BR in promoting cell elongation in various plant species and tissues [24]. However, high concentrations of ECS significantly reduced mature cell length in rice roots. These observations together suggest that endogenous BR content in the elongation zone is closed to saturated for promoting cell elongation, whereas endogenous BR content in the meristem zone is supraoptimal for cell proliferation such that blocking BR biosynthesis led to increased root meristem size. The inhibitory effect of BR on rice root elongation reported here is consistent with previous findings. Upregulation of BR biosynthesis in rice roots by ammonium (NH_4_^+^)-induced miR444-OsBRD1 signaling cascade is responsible for NH_4_^+^-dependent root elongation inhibition [25].

### 3.2. Transcriptional Regulation of Cell Proliferation- and Cell Elongation-Related Genes

Transcriptome analysis showed that ECS significantly repressed *OsPLT1-6* expression, consistent with the strong reduction of meristem size in the ECS-treated roots. Moreover, ECS also repressed expression of several tubulin, kinesin and microtubule-associated proteins, many of which have known function in cell proliferation and elongation [18]. These ECS-repressed kinesin genes included NACK-type kinesin-like protein (*OsNACK*), Gibberellin-deficient dwarf 1 (*OsGDD1*), and Stemless Dwarf 1 (*OsSTD1*), in which mutations led to impaired cell division and elongation [26,27,28]. In Arabidopsis, the BR-activated transcription factors BZR1 and BES1 have been shown to bind the promoter of the microtubule-associated protein *CLASP* gene and repress its expression, resulting in a drastic shift in microtubule organization and a reduction in root meristem cell number [29].

Ethylene has been shown to inhibit primary root elongation in rice by inhibiting root cell proliferation and elongation [30]. Although our results show that the upstream ethylene biosynthesis genes *OsSAMS* and *OsACS* were repressed by ECS, ECS strongly increased transcript of *OsACO1* and *OsACO2* (Figure 4c), which catalyze the final step of ethylene production. Thus, BR may increase ethylene levels, which could contribute to ethylene-mediated root elongation inhibition. Consistently, BR has been shown to increase ethylene production in etiolated rice seedlings through post-transcriptional regulation of ACS proteins [20]. Further research using ethylene signaling mutants or inhibitors of ethylene action is needed to confirm the crosstalk between BR and ethylene in inhibiting rice root elongation.

Inhibition of cell elongation by PPZ treatment was due to reduced expression of most aquaporins and some expansins. On the other hand, inhibition of cell elongation by ECS was due to reduced expression of the majority of aquaporins and cell wall-loosening and remodeling (EXP and XTH) proteins. *EXP, XTH* and aquaporin genes have been shown to be highly expressed in the elongation zone of Arabidopsis roots, and were induced by BR and repressed by auxin [6]. The contrast transcriptional regulation by BR in rice roots, as shown in this study, may be due to complex interaction of BR with other signals that control cell elongation, such as ethylene and gibberellin (GA) [7,30]. Previous research has shown that elevated BR levels or BR signaling inhibit organ growth by promoting expression of the GA- inactivation enzyme GA2ox-3, which reduces bioactive GA levels and cell elongation in rice roots and leaf sheath [7,31].

Taken together, our findings demonstrate the negative effect of high concentrations of BR on cell proliferation and cell elongation-related genes and suggest that optimal BR level in the root is critical for optimal root elongation. A recent study has demonstrated that optimal BR levels are required for root growth, as well as mineral nutrient homeostasis in soybean [32].

### 3.3. Involvement of BR in Low N-Induced Root Elongation

Root growth plasticity in response to nutrient availability is modulated by various phytohormones. Our results showed that N deficiency increased root meristem size, meristem cell number and mature cell length (Figure 5c,d,f), consistent with a previous report [30]. A low concentration of BR treatment (10 nM ECS) inhibited low N-induced root growth, as it reduced root meristem size and meristem cell number but did not block low N-induced cell elongation. On the other hand, PPZ treatment reduced low N-induced root growth, as it reduced low N-induced cell elongation and did not further increase root meristem size and meristem cell number compared to the PPZ-treated roots in normal N condition.

Although results of ECS treatment under normal N condition suggest that endogenous BR level in the roots was optimal and likely saturated for root cell elongation, N deficiency could further promote root cell elongation. It is possible that N deficiency promoted root cell elongation by increasing very low levels of BR in the elongation zone or through non-BR-mediated processes. For instance, a recent work showed that N deficiency reduced cytokinin contents in rice primary roots, resulting in increased root meristem cell proliferation and cell elongation [17]. Further research is needed to quantify endogenous BR levels in the root tips to determine whether N deficiency alters BR contents in the root meristem zone and elongation zone, resulting in increased cell proliferation and elongation.

Patterning of hormonal signals along root developmental axis is critical for optimal root growth and development. Local BR biosynthesis has been shown to peak in the elongation zone [33], coinciding with the optimal onset of cell elongation in the transition zone located between the meristem and elongation zone. As a result, exogenous BR or PPZ treatment may not promote optimal root elongation due to unbalanced activity in the meristem and elongation zone.

## 4. Materials and Methods

### 4.1. Plant Materials and Growth Conditions

Seeds of rice (*Oryza sativa* L.) cv. Look Daeng Pattani, kindly provided by the Pathum Thani rice research center, were used in this study. Seeds were surface-sterilized and germinated in distilled water for 2 d in the dark, before transferring to Yoshida’s nutrient solution (1.427 mM NH_4_NO_3_, 0.323 mM NaH_2_PO_4_, 0.512 mM K_2_SO_4_, 0.998 mM CaCl_2_, 1.643 mM MgSO_4_, 0.009 mM MnCl_2_, 0.075 µM (NH_4_)_6_Mo_7_O_24_, 0.019 mM H_3_BO_3_, 0.152 µM ZnSO_4_, 0.155 µM CuSO_4_ and 0.036 mM Fe-EDTA) [34]. The concentrations of N supply in normal N and low N conditions were 1.427 mM and 0 mM NH_4_NO_3_, respectively. The nutrient solutions were adjusted to pH 5.8 and renewed every 2 d. The seedlings were grown in a growth room at 30 °C with a 12 h/12 h light-dark cycle.

To investigate the effect of PPZ and BR on primary root elongation, germinated rice seeds were grown for 5 d in normal N solution supplemented with various concentrations of PPZ (0, 2, 4, 8 and 16 µM PPZ) or ECS (0, 0.1, 1, 10 and 50 nM ECS) or the combination of 4 µM PPZ and ECS (0, 0.1, 1 and 10 nM ECS). To investigate the effect of PPZ and BR on primary root meristem and transcriptomes, germinated rice seeds were grown for 5 d in normal N media supplemented with or without 4 μM PPZ and then treated with various concentrations of ECS (0, 1, 10, 100 nM and 1 and 10 μM) for 24 h. Only PPZ (4 μM), ECS (10 μM) and mock samples were included in the transcriptomic experiment.

For N deficiency experiments, germinated seeds were grown in normal N solution for 5 d and then transferred to either normal N or low N conditions, which were supplemented with 10 nM ECS or 4 μM PPZ or mock, and continued to grow for 7 d. Root samples were harvested for quantification of crown root length, which was calculated from the average of the three longest crown roots, and crown root meristem.

### 4.2. Chemical Treatment

This study used 24-epicastasterone (ECS), which is a precursor of castasterone, an end product of BR biosynthesis pathway in rice [35], and has been shown to be biologically active but less so than brassinolide [36]. ECS (Yuanye Biology, Shanghai, China), was dissolved in 80% ethanol. Propiconazole (Syngenta, Shanghai, China) was prepared by dissolving in distilled water. For mock treatment, medium with ethanol at the same final concentration as that for ECS treatments was used.

### 4.3. Quantitative Analysis of Root Phenotypes

Root systems were harvested and scanned using a flatbed scanner (EPSON Perfection V850 Pro, Japan), and root length was measured using ImageJ software (https://imagej.nih.gov/ij/). For root meristem quantification, root tips were cut and incubated in a basic solution (7% NaOH in 60% ethanol) for 2 d. Then, the roots were mounted in a solution (50% glycerol in 10% ethanol) [37], and imaged with a microscope (Olympus BX43, Japan). For confocal images of root meristem, the root tips were fixed in fixative (50% methanol and 10% acetic acid) at 4 °C for at least 12 h and then stained using modified pseudo-Schiff propidium iodide (mPS-PI) staining [38]. Briefly, the tissue was incubated in 1% periodic acid, rinsed with water, and then incubated in Schiff reagent (100 mM sodium metabisulphite and 0.15 N HCl) with propidium iodide. The stained samples were mounted on microscope slides with a chloral hydrate solution and visualized on a Zeiss confocal microscope (Carl Zeiss, Oberkochen, Germany).

Root meristem size was determined by measuring the length from the quiescent center (QC) to the first elongated cell in the fourth cortical layer. Meristem cell number and meristem cell length were determined from the number of cells and the average length of all cells in the fourth cortical layer of the root meristem, respectively. Mature cell length was quantified from the average length of five adjacent mature cells in the fourth cortical layer of the root maturation zone, where cells have recently reached their final size within the treatment period. The meristem size and cell length were measured using ImageJ software.

### 4.4. RNA Extraction, cDNA Library Construction and RNA-Seq

For each treatment, three biological replicates (6 plants/ replicate) were included. Total RNA was extracted from root tissues using PureLink RNA Mini Kit based on the manufacturer’s instructions (Invitrogen, Carlsbad, CA, USA) and genomic DNA were removed using DNase I (Thermo Fisher Scientific, Waltham, MA, USA). The quality of the total RNA was assessed by the Agilent 2100 Bioanalyzer (Agilent Technologies, Santa Clara, CA, USA), and all samples had an RNA integrity number (RIN) greater than 8.9. Purification of mRNA, library construction and sequencing were performed at Apical Scientific Sdn. Bhd. (Selangor, Malaysia) using an Illumina NovaSeq 6000 sequencer according to the manufacturer’s instructions (Illumina, San Diego, CA, USA) for 2 × 150 bp paired-end reads. The RNA-seq raw data were deposited in the Sequence Read Archive of the National Center for Biotechnology Information under accession number PRJNA753856.

### 4.5. Data Processing and Bioinformatics Analysis

After pre-processing and filtering low-quality reads, more than 90% of reads could be mapped to the RAPDB reference genome (IRGSP 1.0.21) using HISAT [39] and StringTie [40] pipeline. Comparison of expression profiles was performed using DESeq2 [41]. The significant cutoff for differentially expressed genes was set at adjusted *p*-value < 0.05 and |fold change| > 1.5 (equivalent to |shrunken log_2_fold change| > 0.58).

Gene ontology (GO) enrichment analysis was performed using Plant Regulomics [42] with default parameters set and a threshold false discovery rate (FDR) < 0.02 and plotted by REVIGO [43]. Hierarchical clustering and heatmap analysis were performed using ‘hclust’ and ‘heatmap.2’ packages in R (version 3.5.3). Venn diagram was plotted using DeepVenn (https://www.deepvenn.com). Heatmaps showing expression levels of selected genes were plotted using ClustVis web tools [44].

### 4.6. Statistical Analysis

For quantitation of root length and root meristem, at least ten and six biological replicates were analyzed, respectively. Means and standard deviation (SD) were calculated and analyzed by Student’s *t*-test using IBM SPSS statistics 20.

## 5. Conclusions

Our results show that supraoptimal BR inhibited root meristem size and cell elongation, while PPZ treatment increased root meristem size but also inhibited cell elongation. Transcriptome analysis reveals that ECS and PPZ treatments regulated several genes involved in cell proliferation and cell elongation. Furthermore, the responses of ECS- and PPZ-treated roots under N deficiency show that neither an excess nor an absence of BR could promote the root foraging response. Our findings highlight the crucial roles of optimal BR levels in the rice root meristem for maintaining the balance of cell proliferation and cell elongation to promote root growth.

## Data Availability

The RNA-seq data for all samples are available at the Sequence Read Archive of the National Center for Biotechnology Information under accession number PRJNA753856.

## Supplementary Materials

The following are available online at https://www.mdpi.com/article/10.3390/plants10091962/s1, Table S1: ECS- and PPZ-differentially expressed genes; Table S2: Expression of microtubule-related genes.
